# Chlorpyrifos inhibits neural induction via Mfn1-mediated mitochondrial dysfunction in human induced pluripotent stem cells

**DOI:** 10.1038/srep40925

**Published:** 2017-01-23

**Authors:** Shigeru Yamada, Yusuke Kubo, Daiju Yamazaki, Yuko Sekino, Yasunari Kanda

**Affiliations:** 1 Division of Pharmacology, National Institute of Health Sciences, Tokyo, Japan; 2 Pharmacological Evaluation Institute of Japan (PEIJ), Kanagawa, Japan

## Abstract

Organophosphates, such as chlorpyrifos (CPF), are widely used as insecticides in agriculture. CPF is known to induce cytotoxicity, including neurodevelopmental toxicity. However, the molecular mechanisms of CPF toxicity at early fetal stage have not been fully elucidated. In this study, we examined the mechanisms of CPF-induced cytotoxicity using human induced pluripotent stem cells (iPSCs). We found that exposure to CPF at micromolar levels decreased intracellular ATP levels. As CPF suppressed energy production that is a critical function of the mitochondria, we focused on the effects of CPF on mitochondrial dynamics. CPF induced mitochondrial fragmentation via reduction of mitochondrial fusion protein mitofusin 1 (Mfn1) in iPSCs. In addition, CPF reduced the expression of several neural differentiation marker genes in iPSCs. Moreover, knockdown of *Mfn1* gene in iPSCs downregulated the expression of *PAX6*, a key transcription factor that regulates neurogenesis, suggesting that Mfn1 mediates neural induction in iPSCs. Taken together, these results suggest that CPF induces neurotoxicity via Mfn1-mediated mitochondrial fragmentation in iPSCs. Thus, mitochondrial dysfunction in iPSCs could be used as a possible marker for cytotoxic effects by chemicals.

Growing evidence suggests the involvement of environmental chemicals in neurodevelopmental toxicity, leading to neurobehavioral outcomes such as learning disabilities, attention deficit hyperactivity disorder, cognitive impairment, and autism^1^,^2^. As the fetal brain is inherently more susceptible to chemical-induced toxicity compared to the adult brain, exposure to neurotoxic chemicals during early prenatal period can cause delayed neural disorders at lower doses than in adults^3^,^4^.

Organophosphates, such as chlorpyrifos (CPF), are well known to affect brain structure and neurodevelopmental outcome, resulting in delayed neural disorders^5^,^6^. In regard to this, previous studies using magnetic resonance imaging have shown that prenatal exposure to CPF caused abnormalities in the structure, size, and thickness of cerebral cortex, where was responsible for several higher-order brain functions such as attention, cognition, and emotion^7^. Several reports indicate that CPF causes neurotoxicity in the developing brain of animals. In the developing brain of neonatal rats, CPF exposure impairs neurite outgrowth by inhibiting choline acetyltransferase activity^8^. Maternal exposure to CPF suppresses neurogenesis in the hippocampal dentate gyrus of rat offspring^9^. In addition to *in vivo* effects, there has been reported the cytotoxic effects of micromolar CPF levels *in vitro*. For example, CPF inhibited mitochondrial oxidative phosphorylation^10^ and induced apoptosis in human neuroblastoma SH-SY5Y cells^11^ or human neural precursor cells^12^. As micromolar CPF levels were detected in the blood of human newborns living in an agricultural community^13^, the observations made using micromolar levels of CPF *in vitro* could potentially reflect the biological reactions in a living body. However, the effect of CPF on neurodevelopment has not been precisely elucidated.

Morphological changes of mitochondria are known to contribute to homeostasis^14^,^15^. Under normal circumstances, mitochondria fuses together and forms excessive tubular networks (mitochondrial fusion). These fusion is regulated by fusion factors mitofusin 1 and 2 (Mfn1, Mfn2) and optic atrophy 1 (Opa1)^16^,^17^. In contrast, under stress conditions, mitochondrial networks convert into large numbers of small fragments with spherical and punctate morphology (mitochondrial fission), and are regulated by fission factors, such as fission protein 1 (Fis1) and dynamin-related protein 1 (Drp1)^18^,^19^. This morphological dynamics contributes to the maintenance of mitochondrial functions, including energy generation^14^. Moreover, several studies have shown the relationship between mitochondrial fragmentation and cellular and neurodevelopmental defects. For example, Mfn1 or Mfn2 knockout mice die in midgestation embryo, accompanying with developmental delay. In addition, embryonic fibroblasts from these knockout mice display distinct types of fragmented mitochondria, a phenotype due to a severe reduction in mitochondrial fusion^20^. Thus, Mfn1 is considered to be functionally different from Mfn2. In support to this, Mfn1, not Mfn2, is reported to contribute to Opa1-mediated fusion of mitochondrial inner membrane^16^.

In the present study, we investigated the effect of CPF on neural differentiation using human induced pluripotent stem cells (iPSCs) as a model of human organ development. We focused on the effects of micromolar levels of CPF on mitochondrial dynamics, examining the molecular mechanisms of the process. Our results show that micromolar CPF levels inhibited ATP production through Mfn1 reduction, followed by mitochondrial fragmentation. Moreover, Mfn1-mediated mitochondrial dysfunction suppressed early neural induction by decreasing levels of *PAX6*, a key transcription factor that regulates neurogenesis. These data suggest that CPF-induced neurodevelopmental toxicity is based on impairment of mitochondrial functions in human iPSCs.

## Results

### Effect of CPF on neural differentiation of iPSCs

**Figure 1:**
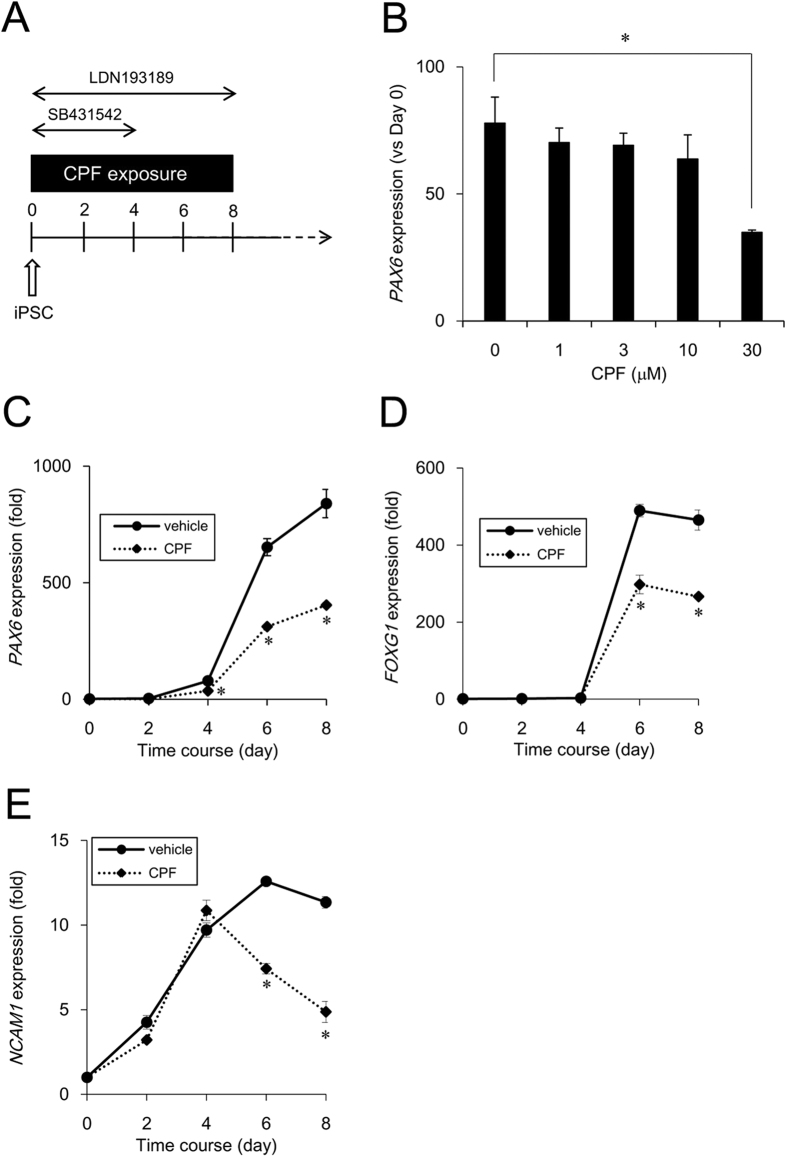
Time course studies of neural induction in iPSCs exposed to CPF. (**A**) Schematic time course of induction from iPSCs to NPCs by dual SMAD inhibition. Neural induction was initiated after exposure to CPF for 24 h. The cells were continuously exposed to CPF throughout neural differentiation. (**B**) At day 4 after neural induction with CPF (0–30 μM), expression of the neural differentiation marker *PAX6* was examined using real-time PCR analysis. (**C–E**) At days 2, 4, 6, and 8 after neural induction with CPF (30 μM), expression of neural differentiation markers, *PAX6, FOXG1*, and *NCAM1* was examined using real-time PCR analysis. Data are represented as means ± SD (n = 3). *P < 0.05.

To investigate whether CPF affects early neurodevelopment, we examined neural differentiation capability of iPSCs, which was induced by dual SMAD inhibition protocol^21^ (Fig. 1A). First, we determined the critical CPF concentration, affecting neural differentiation. At day 4 after neural induction with different concentrations of CPF, the expression of *PAX6*, an early neuroectodermal marker that regulates neurogenesis^22^, was analyzed using real-time PCR. We found that exposure to 30 μM CPF significantly decreased *PAX6* gene expression (Fig. 1B). Next, we performed time course experiments for expression of several neural differentiation markers at days 2, 4, 6, and 8 after exposure to 30 μM CPF. At day 9, almost all cells exposed by CPF (30 μM) were detached from the culture dish. Real-time PCR analysis revealed upregulated expression of *PAX6* by day 4, and *FOXG1*, a neuroectodermal marker that also regulates neurogenesis^23^, thereafter (Fig. 1C and D). Representative neural maturation marker *NCAM1*^24^ continuously increased, confirming that further neural differentiation occurred (Fig. 1E). In addition, CPF exposure reduced the expression of these neural induction markers by day 6 (Fig. 1C–E). These data suggest that CPF has an inhibitory effect on early neural differentiation of iPSCs.

### Mitochondrial function of iPSCs exposed to CPF

**Figure 2:**
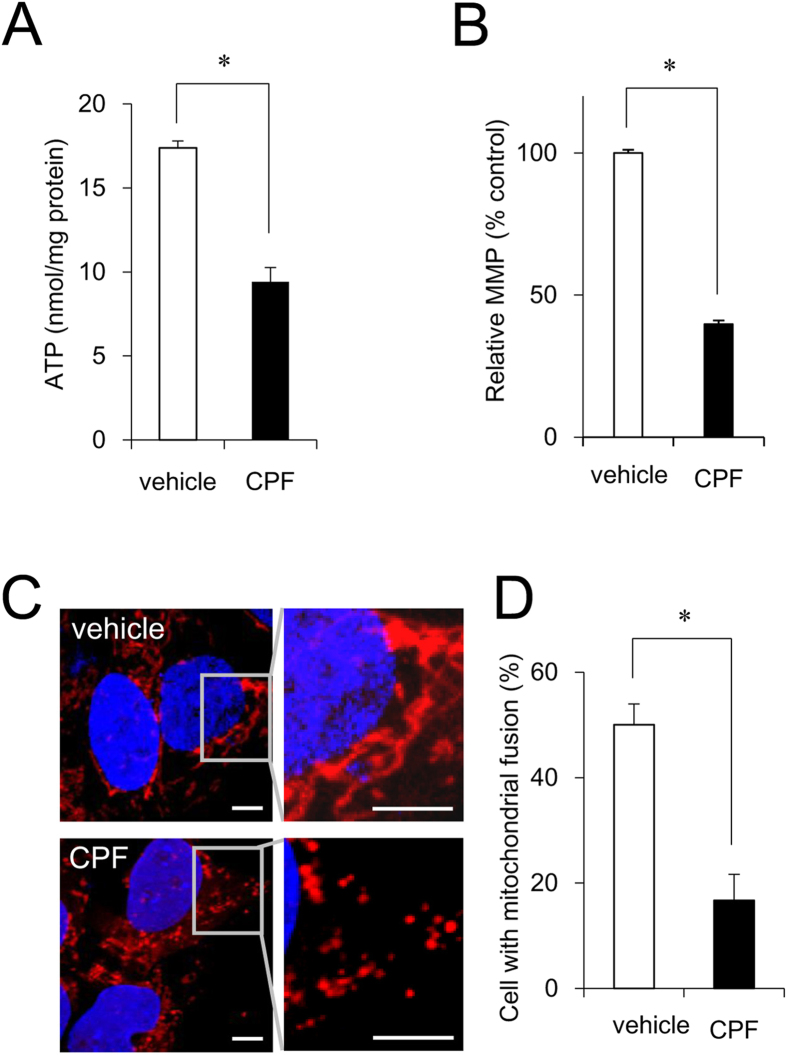
Mitochondrial function of iPSCs exposed to CPF. (**A**) Cells were exposed to CPF (30 μM) for 24 h. Intracellular ATP content was determined in the lysed cells (n = 3). (**B**) Cells were exposed to CPF for 24 h and stained with JC-10 for 20 min. MMP of JC-10 labeled cells was analyzed by flow cytometry. The histogram represents the ratio of JC-aggregate (F-590) to JC-monomer (F-535) fluorescence (n = 3). (**C**) Cells were exposed to CPF for 72 h and stained with MitoTracker Red CMXRos and Hoechst33342. Mitochondrial morphology was observed by confocal laser microscopy. Bar = 5 μm. (**D**) The number of cells with mitochondrial fusion (<10% punctiform) was determined in each image (n = 5). Data are represented as means ± SD. *P < 0.05.

As neural differentiation process requires ATP as a source of energy^25^, we examined intracellular ATP content in iPSCs. Treatment with 30 μM CPF significantly reduced the ATP content of the cells (Fig. 2A). We have previously shown that 0.1 μM carbonyl cyanide m-chlorophenyl hydrazone (CCCP), which functions as a mitochondrial uncoupler^26^, decreased ATP levels in iPSCs. Because CPF inhibited ATP production, we focused on several mitochondrial functions. Mitochondrial membrane potential (MMP) was decreased by exposure to 30 μM CPF for 24 h (Fig. 2B and C). As a positive control, exposure to 0.1 μM CCCP reduced MMP (Figure S1). In addition, CPF exposure increased the number of cells with fragmented mitochondria displaying punctate morphology (Fig. 2D) and decreased the number of cells exhibiting mitochondrial fusion (Fig. 2E). We have already confirmed that 0.1 μM CCCP also increased the occurrence of fragmented mitochondria. These results suggest that CPF induces mitochondrial dysfunction, including MMP depolarization and mitochondrial fragmentation, in iPSCs.

### Expression of mitochondrial fission and fusion factors in iPSCs exposed to CPF

**Figure 3:**
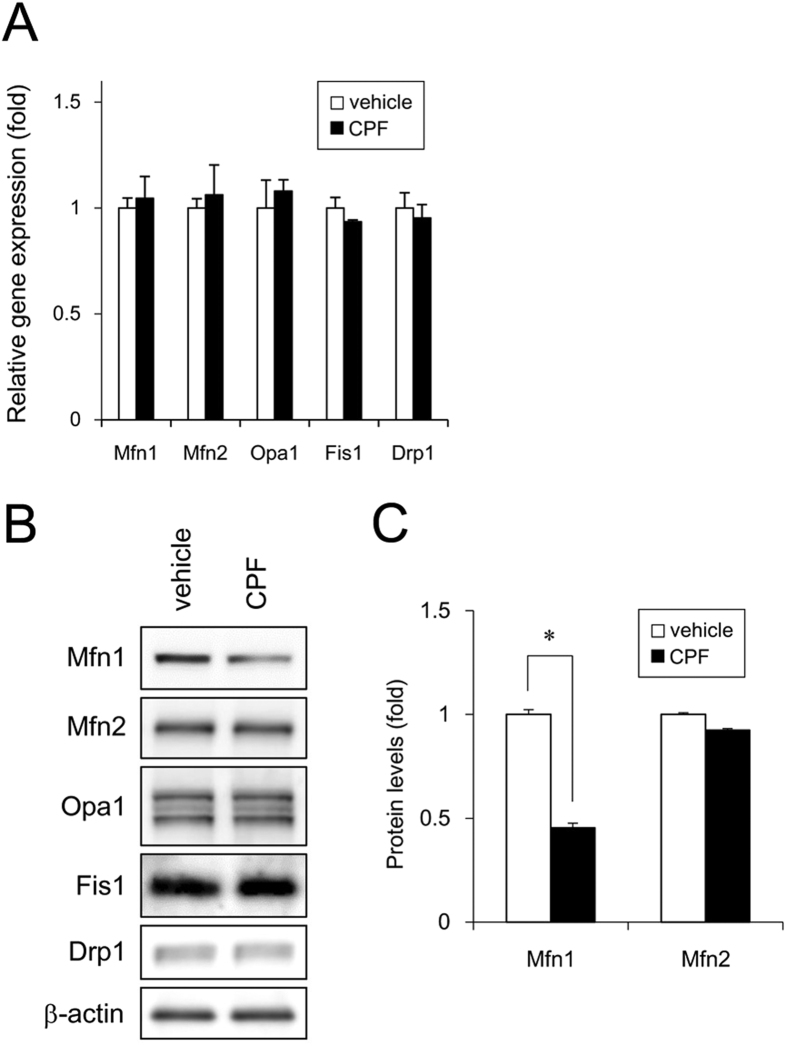
Expression of mitochondrial fission and fusion factors of iPSCs exposed to CPF. (**A**) After exposure to CPF (30 μM) for 24 h, expression of mitochondrial genes was analyzed by real-time PCR. (**B**) After exposure to CPF for 24 h, expression of mitochondrial proteins was analyzed by western blotting using anti-Drp1, anti-Fis1, anti-Mfn1, anti-Mfn2, anti-Opa1, or anti-β-actin antibodies. (**C**) Relative densities of bands were quantified with ImageJ software. Relative changes in expression were determined by normalization to β-actin. Data are represented as means ± SD (n = 3). *P < 0.05.

To examine the molecular mechanisms by which CPF induces mitochondrial fragmentation in iPSCs, we assessed the expression levels of mitochondrial fission (*Fis1* and *Drp1*) and fusion genes (*Mfn1, Mfn2*, and *OPA1*). Real-time PCR analysis showed that the gene expression of the factors was not altered after CPF exposure (Fig. 3A). Interestingly, western blot analysis revealed that CPF significantly decreased Mfn1 protein levels. In contrast, protein expression levels of other factors, including Mfn2, were not changed (Fig. 3B and C). These data suggest that CPF-induced mitochondrial fragmentation is caused by reduction of Mfn1 protein levels.

### Effects of CPF in iPSC-derived neural progenitor cells

To investigate whether the effects of CPF selectively occur in the early stage of neural differentiation in iPSCs, we used iPSC-derived neural progenitor cells (NPCs), which were induced by dual SMAD inhibition protocol^21^ (Figure S1A). Treatment with 30 μM CPF had little effect on ATP content (Figure S1B). Similarly, exposure to 30 μM CPF had little effect on mitochondrial morphology (Figure S1C and D), which was confirmed by the fact that CPF did not alter the protein levels of mitochondrial fission and fusion factors containing Mfn1 (Figure S1E). These data suggest that iPSCs, not NPCs, are sensitive to CPF exposure.

### Effect of Mfn1 knockdown on neural induction of iPSCs

**Figure 4:**
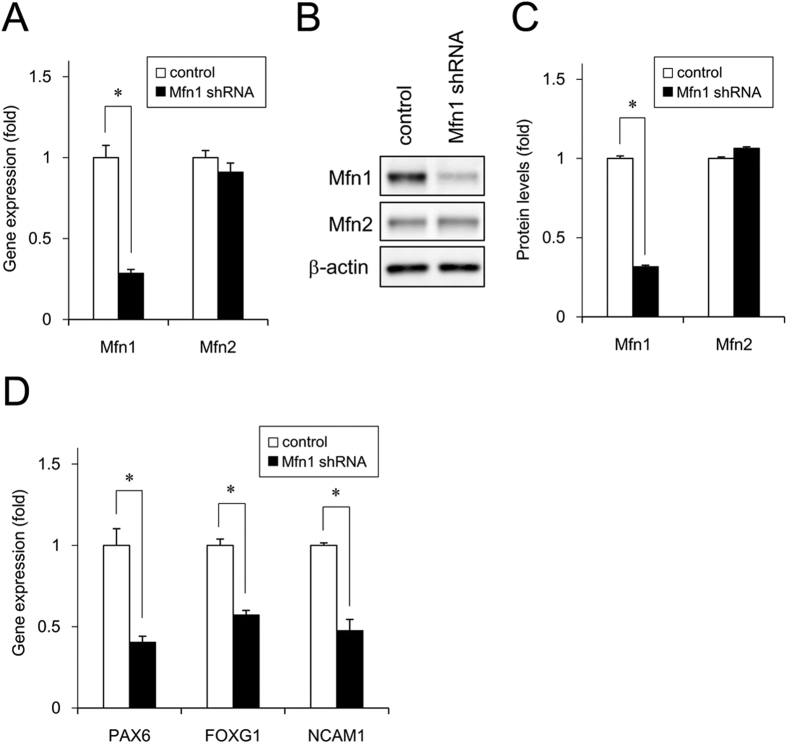
Effect of Mfn1 knockdown on neural induction of iPSCs. Cells were infected with lentiviruses containing a vector encoding a shRNA directed against *Mfn1* or a scrambled sequence shRNA (control) for 24 h. The infected cells were subjected to selection with puromycin (1 μg/ml) for 24 h and cultured for an additional 72 h prior to functional analyses. (**A**) The expression of *Mfn1* and *Mfn2* genes was analyzed by real-time PCR. (**B**) The expression of Mfn1 and Mfn2 proteins was analyzed by western blotting using anti-Mfn1, anti-Mfn2, or anti-β-actin antibodies. (**C**) Relative densities of bands were quantified with ImageJ software. Relative changes in expression were determined by normalization to β-actin. (**D**) Expression of neural differentiation markers *PAX6* (day 4), *FOXG1* (day 6), and *NCAM1* (day 6) was examined with real-time PCR. Data are represented as means ± SD (n = 3). *P < 0.05.

To further investigate the involvement of Mfn1 in the effects of CPF on neural induction, we performed knockdown (KD) of Mfn1, using lentivirus-delivered shRNAs. Real-time PCR analysis showed that KD was selective for *Mfn1*, not *Mfn2*, and that the efficiency was approximately 70% (Fig. 4A). The KD effects were also confirmed by protein levels (Fig. 4B and C). The Mfn1 KD cells were used to perform neural induction. Real-time PCR analysis revealed that Mfn1 KD decreased the expression of *PAX6* (day 4), *FOXG1* (day 6) and *NCAM1* (day 6) (Fig. 4D). These data suggest that Mfn1 is involved in CPF-mediated negative effects on neural induction of iPSCs.

### Negative regulation of neural induction by CPF exposure

**Figure 5:**
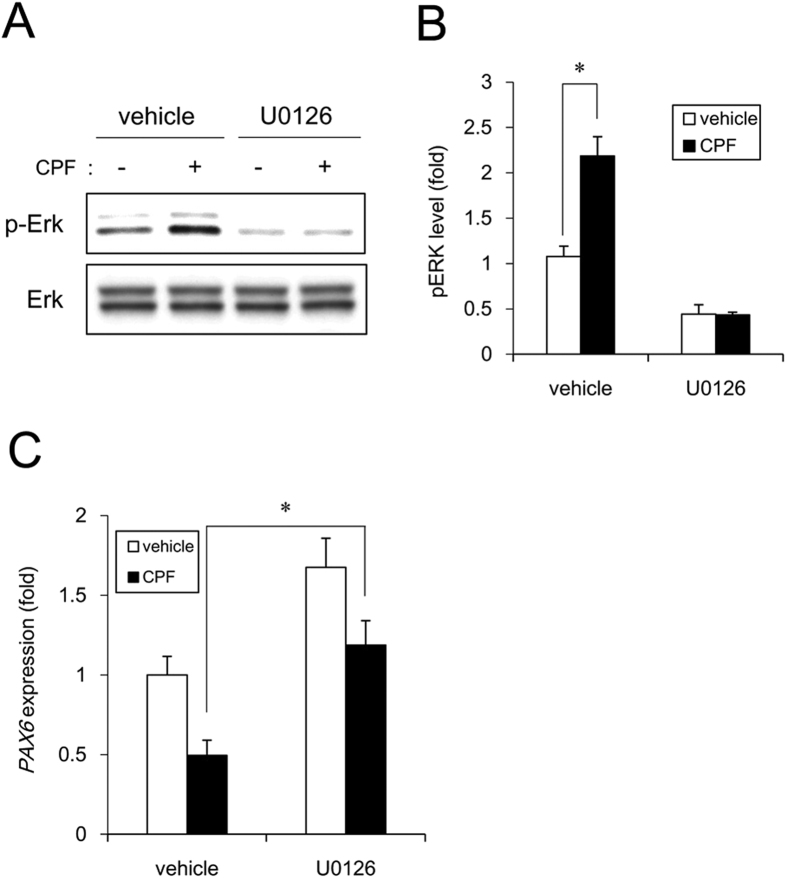
Negative regulation of neural induction by CPF exposure. (**A**) Cells were exposed to CPF (30 μM) or CPF + U0126 (5 μM) for 24 h. ERK phosphorylation was analyzed by western blotting using anti-phospho-ERK antibodies. (**B**) Relative densities of bands were quantified with ImageJ software. Relative changes in expression were determined by normalization to total ERK protein level. (**C**) At day 4 after neural induction with CPF or CPF + U0126, the expression of *PAX6* gene was analyzed by real-time PCR. Data are represented as means ± SD (n = 3). *P < 0.05.

A previous report indicates that ERK signaling inhibits neural induction via *PAX6* silencing in human embryonic stem cells^27^. ERK has been reported to be activated after depletion of Mfn1^28^. We focused on ERK signaling in the effect of CPF on neural induction. We found that CPF exposure significantly increased basal ERK phosphorylation levels, which were abolished by treatment with the ERK inhibitor U0126 (Fig. 5A and B). To further study whether *PAX6* downregulation in CPF-exposed cells occurred through ERK signaling, we examined the effect of U0126 on *PAX6* expression. Incubation with U0126 recovered the expression levels of *PAX6* (Fig. 5C). These data suggest that CPF activates ERK and prevents neural induction via *PAX6* downregulation.

### Effect of Mfn1 knockdown on neural induction

**Figure 6:**
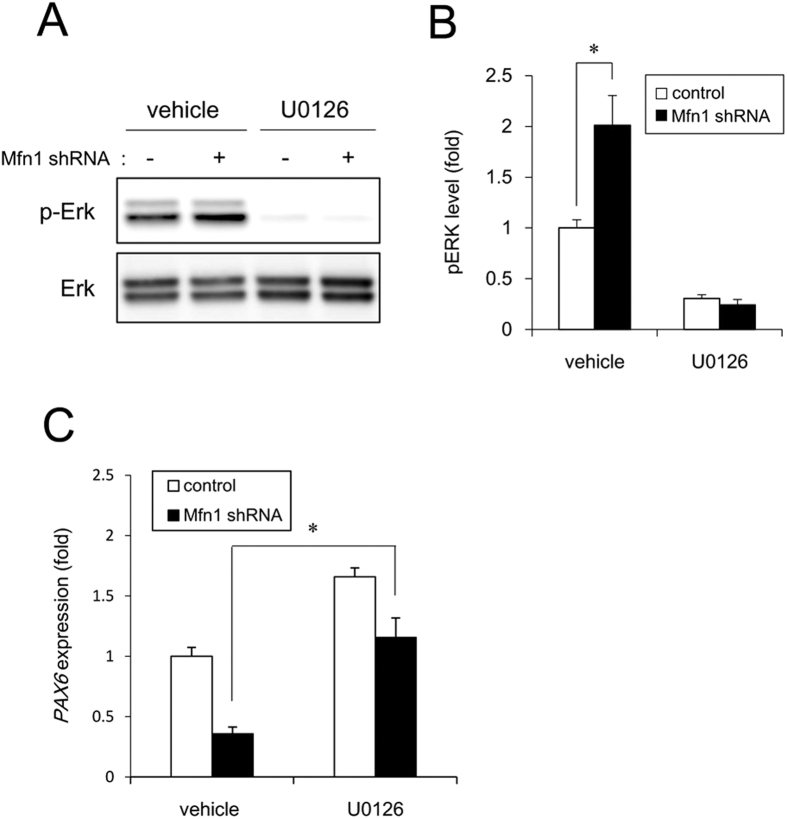
Negative regulation of neural induction by Mfn1 knockdown. The cells were infected with lentiviruses containing a vector encoding a shRNA directed against Mfn1 or a scrambled sequence shRNA (control) for 24 h. The infected cells were subjected to selection with 1 μg/ml puromycin for 24 h and cultured for an additional 72 h prior to functional analyses. (**A**) After incubation with U0126 for 24 h, ERK phosphorylation was analyzed by western blotting using anti-phospho-ERK antibodies. (**B**) Relative densities of bands were quantified with ImageJ software. Relative changes in expression were determined by normalization to total ERK protein level. (**C**) At day 4 after neural induction with U0126, the expression of *PAX6* gene was analyzed by real-time PCR. Data are represented as means ± SD (n = 3). *P < 0.05.

To confirm the involvement of Mfn1 in the inhibition of neural induction by CPF, we used Mfn1 KD cells. Mfn1 KD significantly increased basal ERK phosphorylation levels that were abolished by treatment with the ERK inhibitor U0126 (Fig. 6A and B). To further study whether *PAX6* downregulation in Mfn1 KD cells occurred through ERK signaling, we examined the effect of U0126 on *PAX6* expression. Mfn1 KD decreased *PAX6* by 64% by in the vehicle-treated cells. In contrast, Mfn1 KD decreased *PAX6* by 30% in the U0126-treated cells. Thus, incubation with U0126 partially recovered the *PAX6* expression in the Mfn1 KD cells (Fig. 6C). Taken together, these data suggest that Mfn1 reduction by CPF exposure activates ERK and prevents neural induction via *PAX6* downregulation.

## Discussion

**Figure 7:**
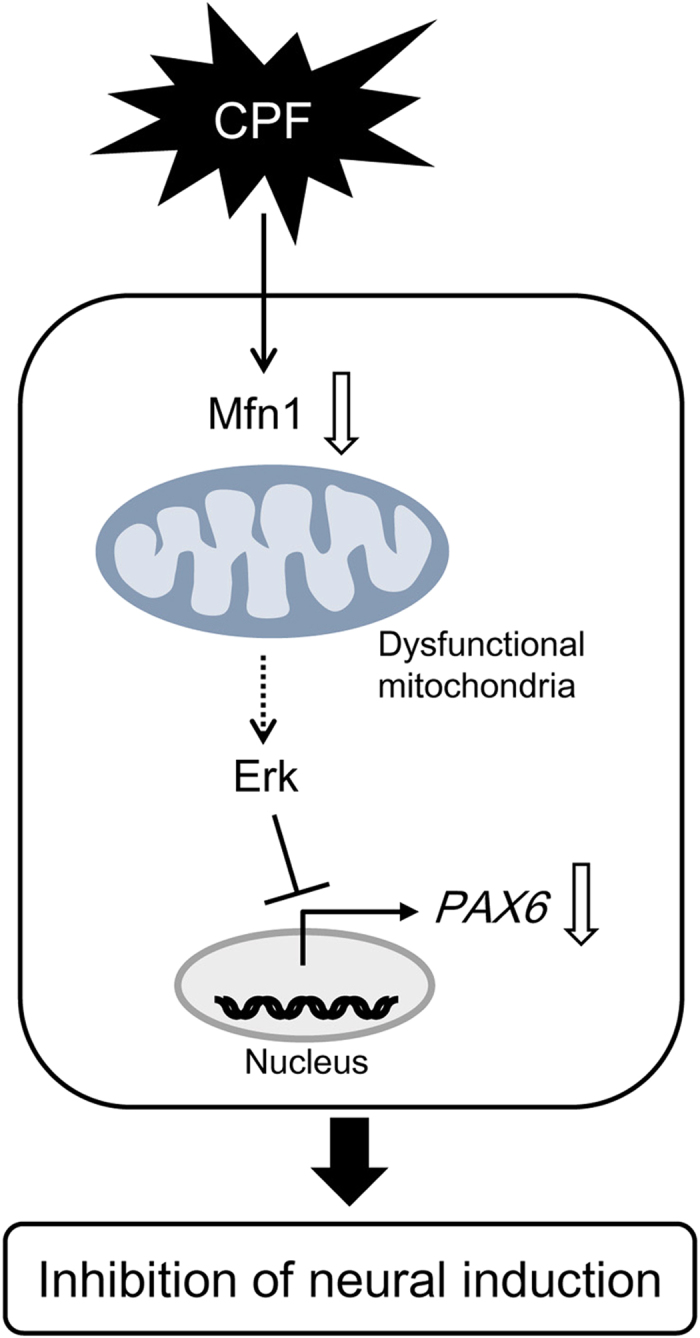
Proposed mechanism of CPF cytotoxicity in human iPSCs. CPF exposure causes Mfn1 reduction, which induces mitochondrial dysfunction, including mitochondrial fragmentation and decreased ATP levels. Mitochondrial dysfunction in turn evokes ERK phosphorylation, leading to the suppression of *PAX6*, which is an early marker of neurogenesis.

In the present study, we demonstrated that exposure to micromolar CPF targeted mitochondrial quality control in human iPSCs. We showed that CPF induced Mfn1 reduction, thereby promoting mitochondrial fragmentation. These negative effects of CPF on mitochondrial quality control could suppress ATP production and neural differentiation. Based on the data observed in our study, Fig. 7 shows a proposed mechanism of CPF cytotoxicity via mitochondrial dysfunction.

Our studies showed that treatment with micromolar CPF levels caused mitochondrial dysfunction of human iPSCs (Fig. 2). We observed that iPSCs were sensitive to CPF exposure, unlike iPSC-derived NPCs (Figure S1). Previous reports support this difference in CPF sensitivity. The inhibitory effect of CPF on DNA synthesis in undifferentiated C6 glioma cells is found to be much higher than in differentiated cells^29^. *In vivo* studies indicate that immature organisms are more susceptible to CPF-induced toxicity compared to adults due to lower levels of CPF metabolizing enzymes^30^. Thus, the difference in CPF sensitivity between iPSCs and NPCs may be dependent on the maturation of CPF detoxification pathways. We are currently conducting experiments to determine the mechanism causing the differences in sensitivity to CPF.

We showed that CPF induced mitochondrial fragmentation via Mfn1 reduction (Figs 2 and 3). Consistent with this, our previous knockdown studies indicated that Mfn1 reduction was sufficient to promote mitochondrial dysfunction^31^. CPF-induced Mfn1 reduction might mediate mitochondrial fragmentation, decrease ATP levels, and inhibit iPSC growth. Although Mfn2 is also involved in mitochondrial fission and energy supply processes^32^,^33^, our results indicated that CPF specifically targeted Mfn1, not Mfn2. Regarding this apparent CPF specificity, E3 ubiquitin ligase membrane-associated RING-CH 5 (MARCH5) has been reported to selectively bind to Mfn1 dependent on its acetylation, and degrade among all mitochondrial proteins, including Mfn2^34^. In addition, we have reported that organotin compounds induced Mfn1 degradation through MARCH5, thereby promoting mitochondrial fragmentation in iPSCs^31^. Thus, CPF may specifically target Mfn1 protein via MARCH5 in iPSCs without affecting mRNA levels. Furthermore, the difference in CPF sensitivity between iPSCs and NPCs may be dependent on Mfn1 and MARCH5 expression levels or MARCH5 activity. Further studies should determine whether CPF reduces Mfn1 via MARCH5-mediated degradation in iPSCs.

We demonstrated that ERK phosphorylation mediated the negative effects of CPF on early neural differentiation (Figs 1, 4 and 5). A previous report indicates that Mfn1 directly binds Ras and Raf, resulting in the inhibition of Ras-Raf-ERK signaling by the biochemical analysis^35^,^36^. Mfn1 reduction by CPF or shRNA may reverse this ERK signaling inhibition. Mobilization of Ca^2+^ from intracellular stores, including mitochondria was reported to result in phosphorylation of MAPKs, as the process was suppressed by chelation of intracellular Ca^2+^ in human T lymphoblastoid cells^37^. As mitochondria are known to uptake into the matrix of any Ca^2+^ that has accumulated in the cytosol, dependent on MMP^38^, mitochondrial dysfunction by CPF exposure may cause an overload of Ca^2+^, resulting in ERK activation. Moreover, ERK signaling was reported to inhibit neural induction by *PAX6* silencing via upregulation of stemness factors *NANOG/OCT4* and downregulation of homeobox transcription factor *OTX2*^27^. NANOG and OCT4 act as repressors of *PAX6* induction, whereas OTX2 is a positive inducer of *PAX6*^27^. Therefore, ERK signaling evoked by CPF could affect the expression of these transcriptional network, including *NANOG, OCT4* and *OTX2, by* regulating *PAX6*. In future studies, we should further investigate the mechanisms of CPF-induced negative regulation of neural induction via ERK.

We further demonstrated that Mfn1 reduction mediated cytotoxic effects of CPF on iPSCs via *PAX6* downregulation (Figs 5 and 6). *FOXG1* was downregulated, along with *PAX6*, during neural differentiation of iPSCs exposed to CPF. *PAX6* and *FOXG1* act as transcriptional regulators during forebrain development in vertebrates^39^,^40^. Targeted disruption of *PAX6* and *FOXG1* in rodents led to the loss of anterior neural tissues, suggesting the central role of these genes in forebrain development^41^,^42^. CPF causes various defects in the development of hippocampus and cortex of rodents^43^. Thus, CPF-induced defects of forebrain architecture may be caused by transcriptional silencing of anterior neural markers during early neurogenesis. As *NCAM1* was downregulated during neural differentiation of iPSCs exposed to CPF, further studies using NPCs are required to reveal how CPF affects neural maturation processes.

In summary, our results demonstrate a novel mechanism underlying cytotoxicity, including neurodevelopmental toxicity of CPF in iPSCs. Recently, significant progress has been made in the induction of differentiation of pluripotent stem cells into a variety of cell types^44^. Further studies are needed to evaluate the developmental effects of CPF on various types of iPSC-derived cells. Moreover, we show that CPF toxicity is caused by Mfn1-mediated mitochondrial dysfunction, which is involved in the cytotoxicity of organotin compounds^31^. Thus, mitochondrial functions influenced by Mfn1 might be a good starting point for investigating toxic mechanisms induced by exposure to other chemicals.

## Methods

### Chemicals

Chlorpyrifos (CPF), Y-27632, SB431542, and LDN193189 were obtained from Wako (Tokyo, Japan). Penicillin-streptomycin mixture (PS) was obtained from Thermo Fisher Scientific (Waltham, MA, USA). U0126 was obtained from Enzo Life Sciences (Farmingdale, NY, USA). Poly-L-ornithine, 2-mercaptoethanol (2-ME), and carbonylcyanide *m*-chlorophenylhydrazone (CCCP) were obtained from Sigma-Aldrich (St. Louis, MO, USA). All other reagents were of analytical grade and obtained from commercial sources.

### Cell culture

Human iPSC line 253G1 (Riken BRC Cell Bank, Tsukuba, Ibaraki, Japan) was established through retroviral transduction of *OCT4, SOX2*, and *KLF4* into adult human dermal fibroblasts^45^. The cells were cultured under feeder-free conditions using human embryonic stem cell (ESC)-qualified Matrigel (BD Biosciences, San Jose, CA, USA) and TeSR-E8 medium (Stemcell Technologies, Vancouver, BC, Canada) at 37 °C in an atmosphere containing 5% CO_2_. For passage, iPSC colonies were dissociated into single cells using Accumax (Innovative Cell Technologies, San Diego, CA, USA) and cultured in TeSR-E8 medium supplemented with Y-27632 (ROCK inhibitor, 10 μM). The NPCs derived from iPSCs were cultured on poly-L-ornithine and Laminin (Thermo Fisher Scientific) coated dishes at 37 °C in an atmosphere containing 5% CO_2_. The culture medium was Neural maintenance medium [NMM; a 1∶1 mixture of DMEM/F12 (Thermo Fisher Scientific) and Neurobasal (Thermo Fisher Scientific) containing N2 (Thermo Fisher Scientific), B27 (Thermo Fisher Scientific), GlutaMAX (Thermo Fisher Scientific), non-essential amino acids (NEAA; Thermo Fisher Scientific), 2-ME, PS]. For passage, NPCs were dissociated into single cells using Accumax and cultured in NMM supplemented with EGF (20 ng/ml), FGF2 (20 ng/ml) and Y-27632.

### Neural differentiation procedure

For the induction of neuronal lineages, dual SMAD inhibition protocol was used as previously described^21^ with modifications. Briefly, iPSC colonies were dissociated into single cells with Accumax. The cells were seeded at a density of 7 × 10^4^ cells/cm^2^ in TeSR-E8 medium on Matrigel-coated plates in order to reach nearly confluent within two days after seeding. The initial differentiation medium was knockout serum replacement (KSR) medium [Knockout DMEM (Thermo Fisher Scientific) containing KSR (Thermo Fisher Scientific), L-glutamine, NEAA, 2-ME, PS] with SB431542 (TGFβ inhibitor, 10 μM) and LDN193189 (BMP inhibitor, 1 μg/ml). After 4 days, N2 medium [Neurobasal containing N2, B27, GlutaMAX, PS] was added to the KSR medium with LDN193189 every two days.

### Measurement of intracellular ATP levels

Intracellular ATP content was measured using an ATP Determination Kit (Thermo Fisher Scientific), according to the manufacturer’s protocol. Briefly, the cells were washed and lysed with 0.1% Triton X-100/PBS. The resulting cell lysates were added to a reaction mixture containing 0.5 mM D-luciferin, 1 mM DTT, and 1.25 μg/mL luciferase and incubated for 30 min at room temperature. Luminescence was measured using a Fluoroskan Ascent FL microplate reader (Thermo Fisher Scientific). The luminescence intensities were normalized to the total protein content.

### Measurement of MMP

A Cell Meter JC-10 Mitochondrial Membrane Potential Assay Kit (AAT Bioquest, Sunnyvale, CA, USA) was used to detect MMP. Briefly, the cells were suspended in staining buffer containing JC-10 and incubated for 20 min at room temperature. After the cells were treated with CPF, a FACS Aria II cell sorter (BD Biosciences) was used to measure the fluorescence intensity ratio, JC-aggregate (F-590)/JC-monomer (F-535).

### Assessment of mitochondrial fusion

After treatment with CPF (30 μM, 72 h), the cells were fixed with 4% paraformaldehyde and stained with 50 nM MitoTracker Red CMXRos (Cell Signaling Technology, Danvers, MA, USA) and 5 μg/mL Hoechst 33342 (Sigma-Aldrich). Changes in mitochondrial morphology were observed using a confocal laser microscope (Nikon A1). Images (n = 5) of random fields were taken, and the number of cells displaying mitochondrial fusion (<10% punctiform) was determined in each image, as previously reported^46^.

### Real-time polymerase chain reaction (PCR)

Total RNA was isolated from iPSCs using TRIzol reagent (Thermo Fisher Scientific), and quantitative real-time reverse transcription (RT)-PCR was performed using a QuantiTect SYBR Green RT-PCR Kit (Qiagen, Valencia, CA, USA) on an ABI PRISM 7900HT sequence detection system (Applied Biosystems, Foster City, CA, USA) as previously reported^47^. Relative changes in transcript levels were normalized to the mRNA levels of glyceraldehyde-3-phosphate dehydrogenase (*GAPDH*). The following primer sequences were used for real-time PCR analysis: *Fis1*, forward, 5′-TACGTCCGCGGGTTGCT-3′ and reverse, 5′-CCAGTTCCTTGGCCTGGTT-3′; *Drp1*, forward, 5′-TGGGCGCCGACATCA-3′ and reverse, 5′-GCTCTGCGTTCCCACTACGA-3′; *Mfn1*, forward, 5′-GGCATCTGTGGCCGAGTT-3′ and reverse, 5′-ATTATGCTAAGTCTCCGCTCCAA-3′; *Mfn2*, forward, 5′-GCTCGGAGGCACATGAAAGT-3′ and reverse, 5′-ATCACGGTGCTCTTCCCATT-3′; *Opa1*, forward, 5′-GTGCTGCCCGCCTAGAAA-3′ and reverse, 5′-TGACAGGCACCCGTACTCAGT-3′; *PAX6*, forward, 5′-ATGTGTGAGTAAAATTCTGGGCA-3′ and reverse, 5′-GCTTACAACTTCTGGAGTCGCTA-3′; *FOXG1*, forward, 5′-GCCACAATCTGTCCCTCAACA-3′ and reverse, 5′-GACGGGTCCAGCATCCAGTA-3′; *NCAM1*, forward, 5′-GGCATTTACAAGTGTGTGGTTAC-3′ and reverse, 5′-TTGGCGCATTCTTGAACATGA-3′; *GAPDH*, forward, 5′-GTCTCCTCTGACTTCAACAGCG-3′ and reverse, 5′-ACCACCCTGTTGCTGTAGCCAA-3′.

### Western blot analysis

Western blot analysis was performed as previously reported^48^. Briefly, the cells were lysed with Cell Lysis Buffer (Cell Signaling Technology). The proteins were then separated by sodium dodecyl sulfate-polyacrylamide gel electrophoresis (SDS-PAGE) and electrophoretically transferred to Immobilon-P membranes (Millipore, Billerica, MA, USA). The membranes were probed with anti-Drp1 monoclonal antibodies (1:1000; Cell Signaling Technology), anti-Fis1 polyclonal antibodies (1:200; Santa Cruz Biotechnology, Santa Cruz, CA, USA), anti-Mfn1 polyclonal antibodies (1:1000; Cell Signaling Technology), anti-Mfn2 monoclonal antibodies (1:1000; Cell Signaling Technology), anti-Opa1 monoclonal antibodies (1:1000; BD Biosciences), anti-ERK1/2 polyclonal antibodies (1:1000; Cell Signaling Technology), anti-phospho ERK1/2 (Thr202/Tyr204) monoclonal antibodies (1:2000; BD Biosciences), and anti-β-actin monoclonal antibodies (1:5000; Sigma-Aldrich). The membranes were then incubated with secondary antibodies against rabbit or mouse IgG conjugated to horseradish peroxidase (Cell Signaling Technology). The bands were visualized using an ECL Western Blotting Analysis System (GE Healthcare, Buckinghamshire, UK). Images were acquired using an LAS-3000 Imager (FUJIFILM, Tokyo, Japan).

### Gene knockdown by shRNA

Knockdown experiments were performed using *Mfn1* shRNA lentiviruses from Sigma-Aldrich (MISSION shRNA), as previously reported^49^. A scrambled hairpin sequence was used as a negative control. Briefly, the cells were infected with the viruses at a multiplicity of infection of 1 in the presence of 8 μg/mL hexadimethrine bromide (Sigma-Aldrich) for 24 h. After medium exchange, the cells were subjected to selection with 1 μg/mL puromycin for 24 h and cultured for an additional 72 h prior to functional analyses.

### Statistical analysis

All data are presented as means ± standard deviation (SD). Analysis of variance (ANOVA) followed by post-hoc Bonferroni test was used to analyze data in Figs 1, 3C, 4, 5, and 6. Student’s t test was used to analyze data in Figs 2, 3A, S1, and S2. *P*-values < 0.05 were considered statistically significant.

## Additional Information

**How to cite this article**: Yamada, S. *et al*. Chlorpyrifos inhibits neural induction via Mfn1-mediated mitochondrial dysfunction in human induced pluripotent stem cells. *Sci. Rep.* **7**, 40925; doi: 10.1038/srep40925 (2017).

**Publisher's note:** Springer Nature remains neutral with regard to jurisdictional claims in published maps and institutional affiliations.

## Supplementary Material

Supplementary Figure
