# Supplementary material for: Chlorpyrifos inhibits neural induction via Mfn1-mediated mitochondrial dysfunction in human induced pluripotent stem cells
Source: Sci Rep. 2017 Jan 23;7:40925. doi: 10.1038/srep40925 (PMC5256306; doi:10.1038/srep40925)
Supplement: Supplementary Figure [file srep40925-s1.pdf]

Supplementary Information

Chlorpyrifos inhibits neural induction via Mfn1-mediated mitochondrial dysfunction in human induced pluripotent stem cells

Shigeru Yamada, Yusuke Kubo, Daiju Yamazaki, Yuko Sekino, and Yasunari Kanda[*]

Division of Pharmacology, National Institute of Health Sciences, Tokyo, Japan

*Correspondence and requests for materials should be addressed to Y.K. (kanda@nihs.go.jp)

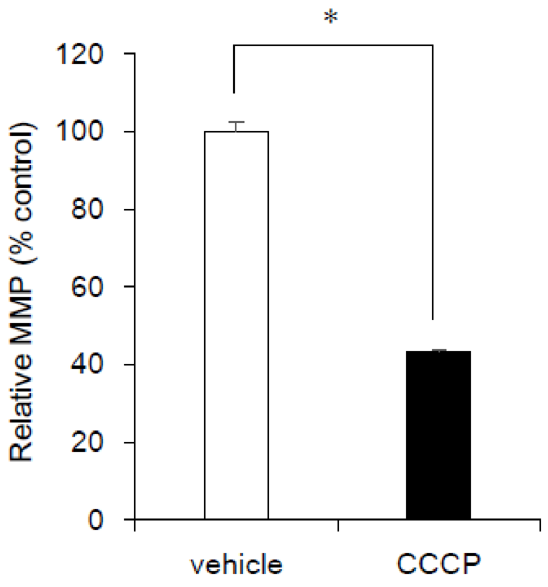

Figure S1. Effects of CCCP on MMP in iPSCs. Cells were exposed to 0.1 μM CCCP for 24 h. Cells were then stained with JC-10 for 20 min. MMP of JC-10 labelled cells was analyzed by flow cytometry. The histogram represents the ratio of JC-aggregate to JC-monomer fluorescence. Data are represented as means ± SD (n = 3). **P* < 0.05.

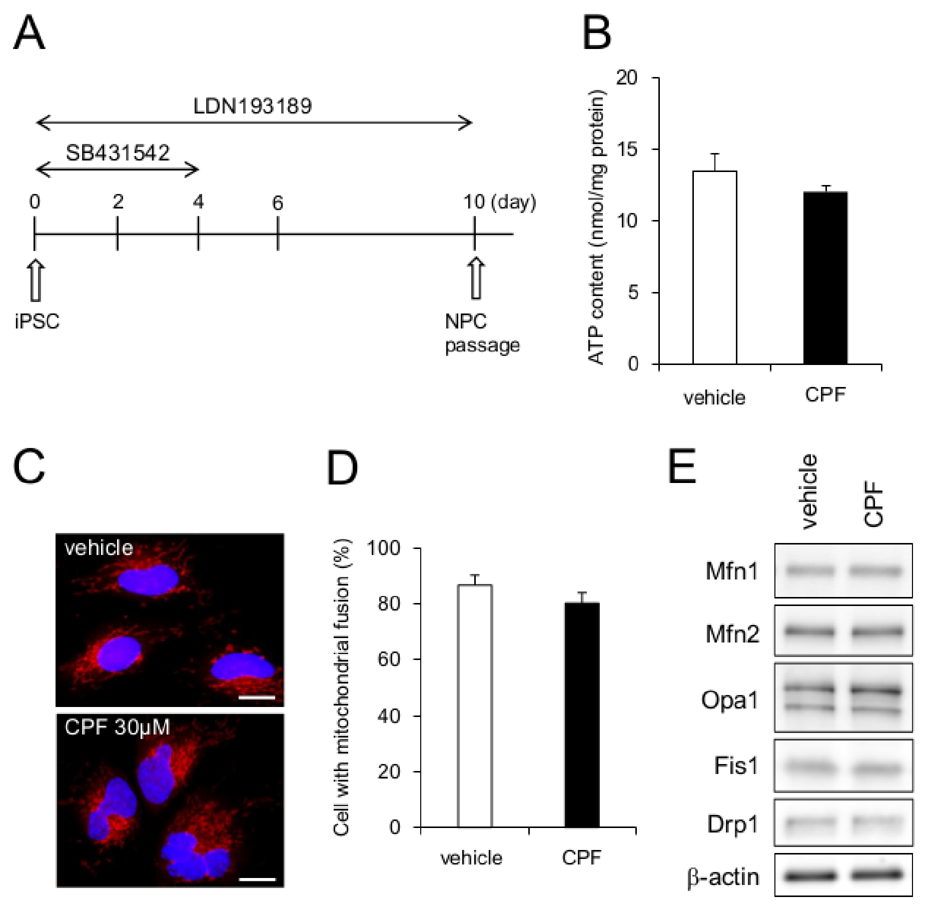

Figure S2. Effects of CPF in iPSC-derived NPCs. (A) Schematic time course of induction from iPSCs to NPCs by dual SMAD inhibition. (B) Cells were exposed to 30 μM CPF for 24 h. The intracellular ATP content was determined in the lysed cells (n = 3). (C) Cells were exposed to 30 μM CPF for 72 h. Cells were then stained with MitoTracker Red CMXRos and Hoechst33342. Mitochondrial morphology was observed by confocal laser microscopy. Bar = 10 μm. (D) The number of cells with mitochondrial fusion (< 10% punctiform) was counted in each image (n = 5). (E) After exposure to 30 μM CPF for 24 h, the expression of mitochondrial proteins was analyzed by western blotting using anti-Drp1, anti-Fis1, anti-Mfn1, anti-Mfn2, anti-Opa1, or anti-β-actin antibodies. Data are represented as means ± SD. *P < 0.05.
